# Clinical-pathological features and perioperative outcomes of mediastinoscopy vs. thoracoscopy esophagectomy in esophageal cancer: A meta-analysis

**DOI:** 10.3389/fsurg.2023.1039615

**Published:** 2023-02-14

**Authors:** Sheng Gong, Xin Rao, Ye Yuan, Xiaojun Yao, Gang Li, Ning Wang, Dan Li, Liangshuang Jiang

**Affiliations:** ^1^ Department of Thoracic Surgery, The Public Health Clinical Center of Chengdu, Chengdu, China; ^2^ Department of Public Health, Chengdu Medicine College, Chengdu, China

**Keywords:** esophageal cancer, mediastinoscopy, thoracoscopy, esophagectomy, perioperative outcomes

## Abstract

**Objective:**

To compare the clinicopathological features and perioperative outcomes of video-assisted mediastinoscopy esophagectomy (VAME) compared to video-assisted thoracoscopy esophagectomy (VATE) in esophageal cancer.

**Methods:**

We comprehensively searched online databases (PubMed, Embase, Web of Science and Wiley online library) to find available studies exploring the clinicopathological features and perioperative outcomes between VAME and VATE in esophageal cancer. Relative risk (RR) with 95% confidence interval (CI) and standardized mean difference (SMD) with 95% CI were used to evaluate the perioperative outcomes and clinicopathological features.

**Results:**

A total of seven observational studies and one randomized controlled trial involving 733 patients were considered eligible for this meta-analysis, of which 350 patients underwent VAME in contrast to 383 patients underwent VATE. Patients in the VAME group had more pulmonary comorbidities (RR = 2.18, 95% CI 1.37–3.46, *P* = 0.001). The pooled results showed that VAME shortened the operation time (SMD = −1.53, 95% CI −2.308–−0.76, *P* = 0.000), and retrieved less total lymph nodes (SMD = −0.70, 95% CI −0.90–−0.50, *P* = 0.000). No differences were observed in other clinicopathological features, postoperative complications or mortality.

**Conclusions:**

This meta-analysis revealed that patients in the VAME group had more pulmonary disease before surgery. The VAME approach significantly shortened the operation time and retrieved less total lymph nodes and did not increase intra- or postoperative complications.

## Introduction

Esophageal cancer is the eighth most frequently diagnosed cancer worldwide accounting for millions of deaths each year due to its poor prognosis especially in Asian countries (1, 2). Surgery plays a substantial role in treating esophageal cancer, with the rapid development of neoadjuvant and adjuvant therapies (3, 4). During the past few decades, minimally invasive surgery has gained steady progress in the field of esophagectomy, and minimally invasive esophagectomy could achieve equal or better oncologic outcomes (5, 6). Minimally invasive esophagectomy has become the chief choice in many institutions.

Traditional minimally invasive esophagectomy releases the esophagus through the thoracic cavity, known as video-assisted thoracoscopy esophagectomy (VATE) (7). In this operation, unilateral pulmonary ventilation cessation or carbon dioxide artificial pneumothorax is imperative to make adequate space for operation, which inevitably narrow the surgical indications, particularly for elderly patients or those with poor cardiopulmonary function. The novel minimally invasive esophagectomy, video-assisted mediastinoscopy esophagectomy (VAME), in which the thoracic segment of the esophagus is released through the posterior mediastinum under direct vision with the assistance of mediastinoscopy, without interrupting the breath and oxygenation during the operation, hopefully reducing trauma and gives operation chance for those who could not put up with oxygenation reduction, particularly for those with poor cardiopulmonary function (8, 9).

Since the introduction of VAME, surgeons focused on this field have attempted to apply this technology to appropriate patients. Case series and cohort studies have been reported, while the perioperative results were not consistent or even opposed in certain outcomes (10–25), such as operation time, lymph node retrieval or postoperative complications. Considering that only a limited number of studies with small sample size have been conducted to compare the superior and inferior of VAME and VATE, it is reasonable to perform a meta-analysis to pool the results from published studies to provide relatively valid evidence and conclusions.

## Materials and methods

### Literature search and selection

A systematic and comprehensive literature search of the online databases PubMed, Embase (*via* OVID), Web of Science and Wiley online library was performed to identify potential studies published before November 23, 2021 that explored the perioperative outcomes as well as clinicopathological features in esophageal cancer patients who received VAME compared to those received VATE. References of the included studies were manually reviewed to identify additional potential available studies. Key words and related variants were used in the search, including esophageal cancer, esophageal neoplasm, video-assisted, mediastinoscopy, thoracoscope, etc. The searching strategy was included as supplementary material. We evaluated all searched results according to the Preferred Reporting Items for Systematic Reviews and Meta-Analyses (PRISMA) (5) guidelines.

### Study inclusion/exclusion criteria

Studies satisfying the following criteria were considered eligible for this meta-analysis.

Inclusion criteria: randomized controlled trials (RCTs) or observational studies that investigated the clinical effectiveness of VAME compared with VATE; one or more interest outcomes were reported: operation time, retrieved lymph nodes, intraoperative blood loss, postoperative complications, mortality, duration of postoperative hospitalization; only studies reported in English were included.

Exclusion criteria: studies without interested parameters including noncomparative studies, reviews, abstracts, case or series reports, new technical studies and letters, robot-assisted surgery was also considered ineligible.

### Definition of VAME

The patient was placed in the supine position with bilateral lung ventilation. An incision was made through the left neck, and the cervical surgery team performed upper and middle esophageal mobilization with the video-assisted mediastinoscope *via* the left cervical approach. The cervical esophagus should be exposed carefully to preserve the recurrent laryngeal nerve. Care must be taken to avoid any damage to the membrane of trachea and main bronchus when dividing the area of the tracheal bifurcation. The abdominal surgery team performed the lower esophageal and gastric dissection *via* a transabdominal approach either simultaneously or subsequently.

### Data extraction and quality assessment

Data were extracted independently by two investigators, and conflicts were adjudicated by team discussion. The following outcomes were used to compare the two surgical methods: operation time, lymph nodes retrieved, intraoperative blood loss, postoperative complications, mortality, and duration of postoperative hospitalization. Available clinicopathological features were also compared.

The Cochrane handbook risk of bias (RoB2—2019) was used to assess the risk of bias in RCTs. Newcastle-Ottawa Scale (NOS) was employed to assess the quality level of non-randomized studies (26). The NOS contains three items: patient selection, comparability of the study groups and assessment of outcome. A high-quality study was defined as a study with quality scores ≥7 (Table 1). Any disagreement was resolved *via* team discussion.

**Table 1:** Basic characteristics of included studies.

Authors	Publishing year	Country	Study period	Sample size (VAME/VATE)	Age (years) (MAE/ TAE)	TNM stage	Pathology			NOS	Study design
							ESCC	EAC	Other		
Koide N et al	2011	Japan	1997–2009	17/37	Mean:66.3 ± 12.9/ 65.3 ± 8.9	I/II or more	49	0	5	8	ROS
Feng MX et al	2011	China	2000–2009	27/27	Median:58.6 (37–79)/61.1 (46–76)	0-IV	54	0	0	8	Pair-matched case–control study
Nomura T et al	2016	Japan	2001–2005	20/15	Mean:64/65	NA	NR			6	ROS
Wang QY et al	2014	China	2005–2010	109/58	Median:62 (54–78)/62 (55–72)	T1	167	0	0	5	ROS
Jin YX et al	2018	China	2016–2017	19/30	Mean:62.50 ± 8.46/59.74 ± 7.92	I–IIIB	48	1	0	6	ROS
Guo L et al	2020	China	Jun 2015 -Jan2019	28/48	Mean: 66.71 ± 8.10/ 63.69 ± 6.03	0 -IIIc -	76	0	0	7	Retrospective case-control study
Liu W et al	2020	China	Jan 2018 to Dec 2019	30/68	Mean: 58.03 ± 8.79/56.97 ± 8.88	cT1-N0-1M0	98	0	0	8	ROS
Shi KF et al	2021	China	NA	100/100	66.3 ± 6.7/66.3 ± 6.1	I–III	200	0	0	NA	RCT

VAME, video-assisted mediastinoscopy esophagectomy; VATE, video-assisted thoracoscopic esophagectomy; ESCC, esophageal squamous cell carcinoma; EAC, esophageal adenocarcinoma; NOS, Newcastle-Ottawa Scale; NR, not reported; ROS, retrospective observational study; RCT, randomized controlled trial.

### Statistical analysis

The relative ratio (RR) and standardized mean difference (SMD) with 95% CIs were calculated for categorical data and continuous data respectively. We used the Cochran chi-square test and *I*^2^ to examine the heterogeneity among studies. Statistical heterogeneity among studies was defined as an *I*^2^ statistic greater than 50%. A fixed-effects model was preferred to a random-effects model when there was no statistically significant heterogeneity. We planned to perform and examine a funnel plot, as well as Begg's test and Egger's test to explore possible publication biases (27). However, we would not produce any funnel plots if the number of researches included was less than 10. Statistical significance was taken as 2-sided (*P* < 0.05). Theanalysis was conducted with STATA 14.0 software (Stata Corporation, College Station, TX).

## Results

### Study selection

**Figure 1:**
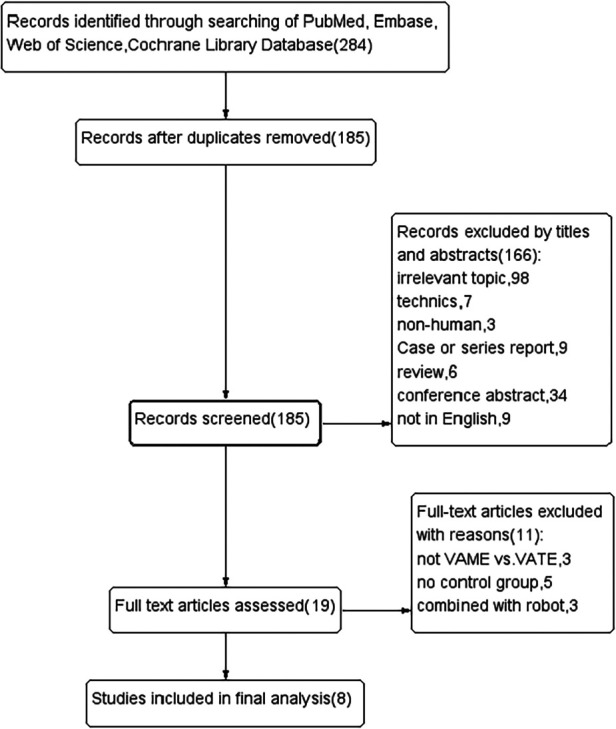
Flow diagram of selecting studies.

Records were screened from previously mentioned online databases. A manual search and inspection of the reference lists identified no additional relevant studies. After exclusion of duplications, a total of 185 studies remained. Then 166 records were immediately excluded by screening the titles and abstracts. We read the full text of the remaining 19 studies carefully, and 8 studies meeting our criteria were finally considered eligible in this meta-analysis (18–25). The ﬂow chart of the literature evaluation process in our meta-analysis is presented in Figure 1.

### Baseline characteristics of the included studies

Six of eight studies were conducted in China and another two were conducted in Japan. Seven studies were retrospective observational studies, of which one was a pair-matched case-control study, and the other one was a RCT. Data from a total of 733 patients were recorded, of which 350 patients underwent VAME in contrast to 383 patients underwent VATE. Patients in the VATE group received thoraco-laparoscopic three-incision esophagectomy, namely, the McKeown esophagectomy, while patients in the VAME group received mediastinoscopy combined laparoscopy or laparotomy esophagectomy. The main data extracted from the included studies are presented in Table 1.

### Quality assessment

**Figure 2:**
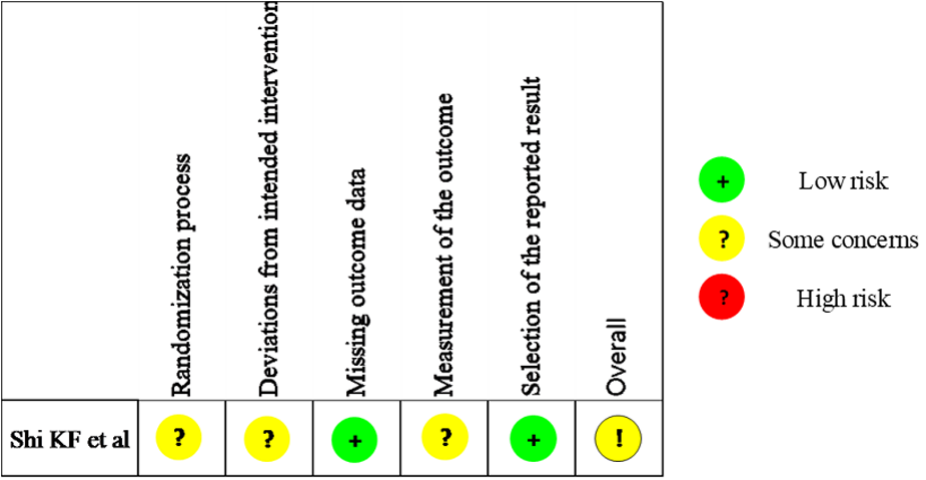
Risk of bias assessment of the included randomized controlled trial.

Quality assessment results of the observational studies were depicted in Table 1 and the summary figure of the RCT was depicted in Figure 2. Four out of the seven observational studies were ranked with medium quality (18, 20, 22, 24), while the other three were ranked with high quality (19, 21, 25) (Table 1). The RCT arose some concerns regarding the risk of bias (23).

### Clinical-pathological features

**Figure 3:**
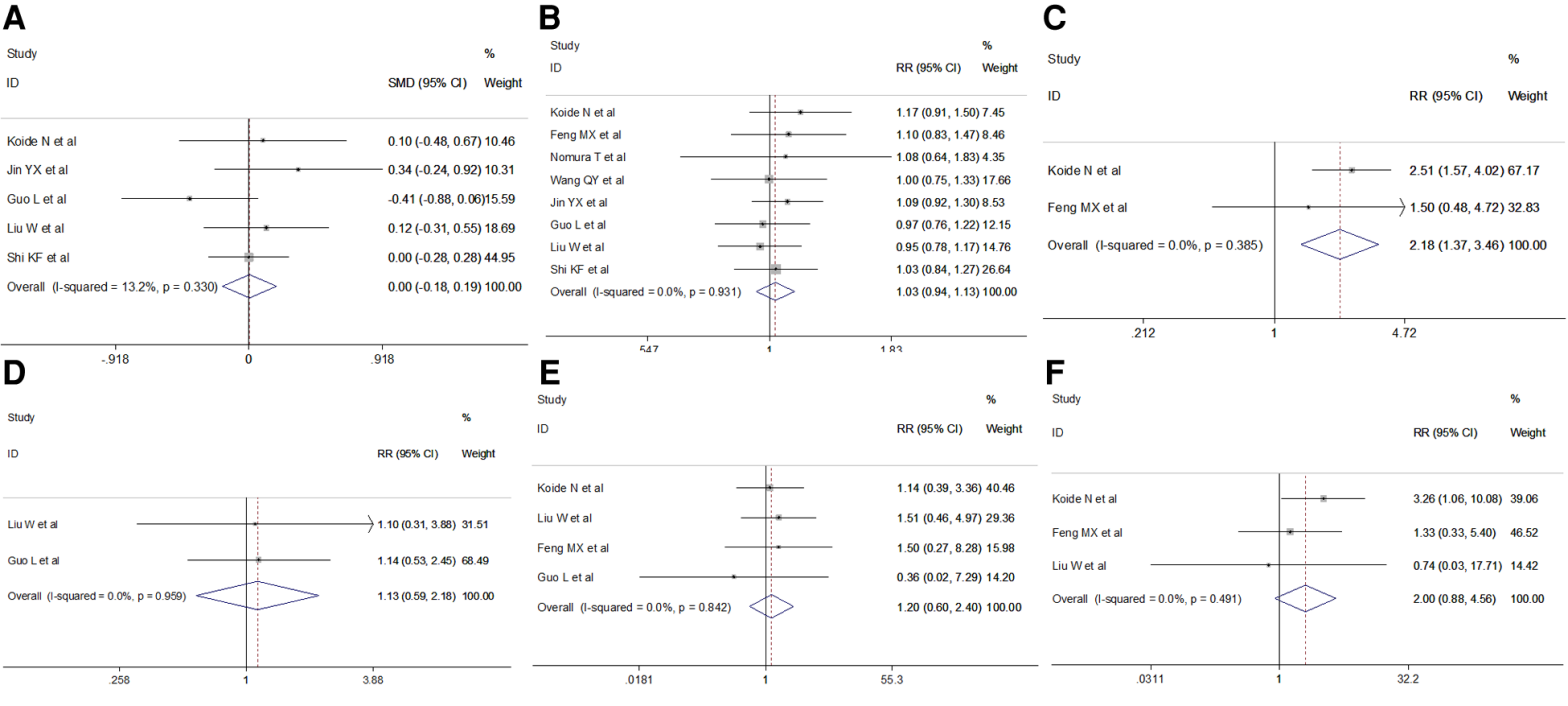
Meta-analysis of clinical features: (**A**) Age; (**B**) Gender; (**C**) Pulmonary disease; (**D**) Hypertension; (**E**) Diabetes; (**F**) Cardiac disease.

Clinical parameters, including age, sex, comorbidities and pathology parameters including pathological type, tumor stage and tumor location were obtained. The pooled results revealed no significant difference in age (fixed effect: SMD = 0.00, 95% CI −0.18–0.19, *P* = 0.966; *I*^2 ^= 13.2%) or sex (fixed effect: RR = 1.03, 95% CI 0.94–1.13, *P* = 0.546; *I*^2 ^= 0%) in the VAME group compared to the VATE group. Patients in the VAME group had more pulmonary disease (fixed effect: RR = 2.18, 95% CI 1.37–3.46, *P* = 0.001; *I*^2 ^= 0%), but not other comorbidities including hypertension (fixed effect: RR = 1.13, 95% CI 0.59–2.18, *P* = 0.716; *I*^2 ^= 0%), diabetes (fixed effect: RR = 1.20, 95% CI 0.60–2.40, *P* = 0.612; *I*^2 ^= 0%) and cardiac disease (fixed effect: RR = 2.00, 95% CI 0.88–4.56, *P* = 0.098; *I*^2 ^= 0%) (Figure 3, Table 2).

**Table 2:** Meta-analysis of clinical-pathological features.

Analysis item	No. of studies	Effects model	RR/SWD (95% CI)	Significance	Heterogeneity test		
					Chi^2^	*I* ^2^	*P*
Age	5	Fixed	SMD = 0.00, 95% CI −0. 18–0.19	*P* = 0.966	4.61	13.2	0.330
Sex	8	Fixed	RR = 1.03, 95% CI 0.94–1.13	*P* = 0.546	2.45	0	0.931
Hypertension	2	Fixed	RR = 1.13, 95% CI 0.59–2.18	*P* = 0.716	0	0	0.959
Pulmonary disease	2	Fixed	RR = 2.18, 95% CI 1.37–3.46	*P* = 0.001	0.76	0	0.385
Diabetes	4	Fixed	RR = 1.20, 95% CI 0.60–2.40	*P* = 0.612	0.83	0	0.842
Cardiac disease	3	Fixed	RR = 2.00, 95% CI 0.88–4.56	*P* = 0.098	1.42	0	0.491
Tumor length	3	Fixed	SMD = −0.02, 95% CI −0.45–0.40	*P* = 0.917	0.01	0	0.749
Pathology (ESCC vs others)	4	Fixed	RR = 1.04, 95% CI 0.94–1.14	*P* = 0.432	3.57	16.1	0.311
Overall stage (II–IV vs 0–I)	6	Fixed	RR = 0.98, 95% CI 0.88–1.10	*P* = 0.720	1.01	0	0.908
Tumor location (cervical/upper thoracia vs middle thoracic/lower thoracic/ abdominal esophagus)	5	Fixed	RR = 0.88, 95% CI 0.59–1.30	*P* = 0.513	1.30	0	0.861

RR, relative risk; SMD, standardized mean difference; CI, confidence interval; ESCC, esophageal squamous cell carcinoma.

**Figure 4:**
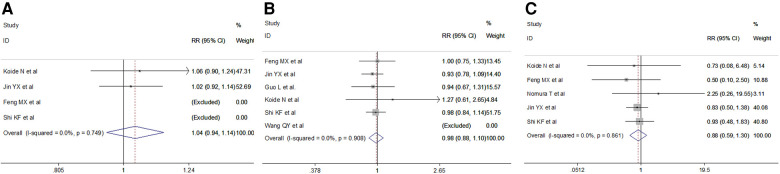
Meta-analysis of pathological features: (**A**) Pathology; (**B**) Stage; (**C**) Tumor location.

No differences were observed in pathological type (fixed effect: RR = 1.04, 95% CI 0.94–1.14, *P* = 0.432; *I*^2 ^= 16.1%) in the VAME group compared with the VATE group. The pooled results indicated no difference regarding tumor stage in the VAME group (fixed effect: RR = 0.98, 95% CI 0.88–1.10, *P* = 0.7204; *I*^2 ^= 0%) or tumor location (fixed effect: RR = 0.88, 95% CI 0.59–1.30, *P* = 0.513; *I*^2 ^= 0%) compared to the VATE group (Figure 4, Table 2).

### Intraoperative outcomes

**Figure 5:**
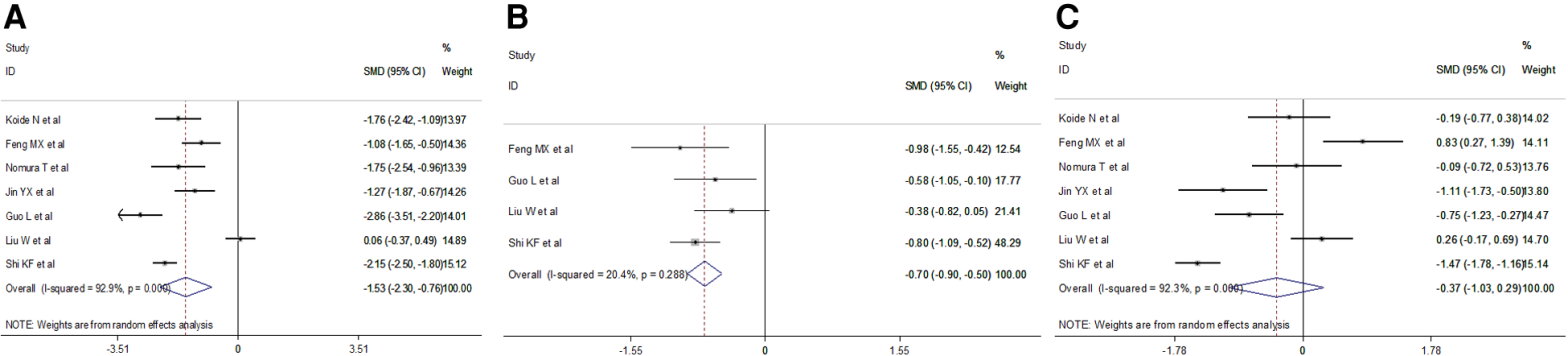
Meta-analysis of intraoperative outcomes: (**A**) Operation time; (**B**) Total lymph node retrieved; (**C**) Intraoperative blood loss.

We retrieved intraoperative data including operation time, intraoperative blood loss and total lymph nodes retrieved. Meta-analysis results indicated a shorter operation time (random effect: SMD = −1.53, 95% CI −2.308–−0.76, *P* = 0.000; *I*^2 ^= 92.9%) and less total lymph nodes (fixed effect: SMD = −0.70, 95% CI −0.90–−0.50, *P* = 0.000; *I*^2 ^= 20.4%) in the VAME group, but no difference in intraoperative blood loss (random effect: SMD = −0.37, 95% CI −1.03–0.29, *P* = 0.275; *I*^2 ^= 92.3%) compared to the VATE group (Figure 5, Table 3).

**Table 3:** Meta-analysis of perioperative outcomes.

Analysis item	No. of studies	Effects model	RR/SWD (95% CI)	Significance	Heterogeneity test		
					Chi^2^	*I* ^2^	*P*
Operation time	7	Random	SMD = −1.53, 95% CI −2.30–−0.78	*P* = 0.000	84	92.9	0.000
Intraoperative hemorrhage	7	Random	SMD = −0.37, 95% CI −1.03–0.29	*P* = 0.275	77.65	92.3	0.000
Number of lymph nodes retrieved	4	Fixed	SMD = −0.70, 95% CI −0.90–−0.50	*P* = 0.000	3.77	20.4	0.288
Postoperative hospital stay	4`	Random	SMD = −0.21, 95% CI −0.72–0.31	*P* = 0.434	12.57	76.1	0.006
Morbidity							
Laryngeal recurrent nerve damage	7	Random	RR = 2.24 95% CI 0.93–5.39	*P* = 0.071	12.36	51.5	0.054
Anastomotic leakage	7	Fixed	RR = 1.02, 95% CI 0.69–1.51	*P* = 0.927	2.57	0	0.861
Pulmonary complications	3	Random	RR = 0.80, 95% CI 0.34–1.86	*P* = 0.349	5.26	62.0	0.072
Pneumonia	5	Random	RR = 0.54, 95% CI 0.15–1.90	*P* = 0.335	22.92	82.5	0.000
Chylothorax	7	Fixed	RR = 0.34, 95% CI 0.11–1.02	*P* = 0.055	0.39	0	0.996
Arrhythmia	3	Fixed	RR = 1.17, 95% CI 0.38–3.64	*P* = 0.783	0.36	0	0.635
Mortality	6	Fixed	RR = 0.75, 95% CI 0.16–3.58	*P* = 0.722	1.52	0	0.468

RR, relative risk; SMD, standardized mean difference; CI, confidence interval.

### Postoperative outcomes

**Figure 6:**
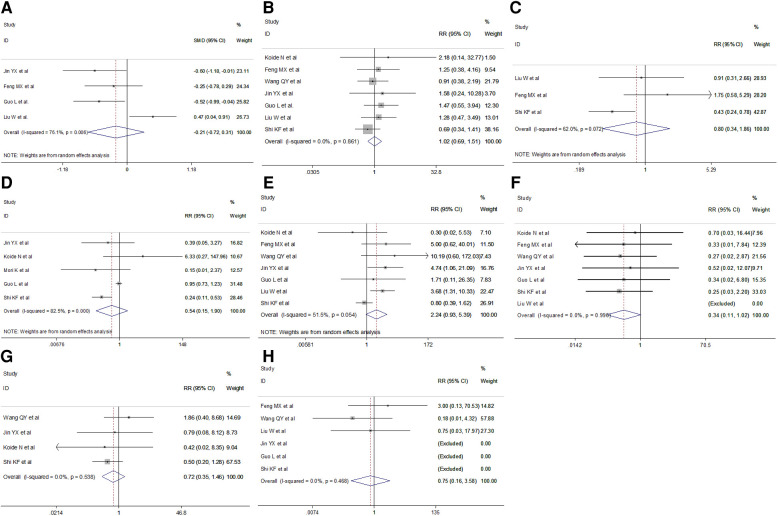
Meta-analysis of postoperative outcomes: (**A**) Postoperative hospital stay; (**B**) Anastomotic leakage; (**C**) Respiratory complications; (**D**) Postoperative pneumonia; (**E**) Laryngeal recurrent nerve injury; (**F**) Chylothorax; (**G**) Arrhythmia; (**H**) Mortality.

Short-term postoperative outcomes for analysis included length of postoperative hospital stay and specific complications such as laryngeal recurrent nerve injury, anastomotic leak, postoperative pneumonia, chylothorax, arrhythmia and mortality. Meta-analysis indicated no difference in the duration of postoperative hospital stay in the VAME group compared with the VATE group (random effect: SMD = −0.21, 95% CI −0.72–0.31, *P* = 0.434; *I*^2 ^= 76.1%). No differences were observed regarding postoperative complications including anastomotic leakage(fixed effect: RR = 1.02, 95% CI 0.69–1.516, *P* = 0.404; *I*^2 ^= 0%), postoperative pulmonary complications (random effect: RR = 0.80, 95% CI 0.34–1.86, *P* = 0.050; *I*^2 ^= 62.0%), pneumonia(random effect: RR = 0.54, 95% CI 0.15–1.90, *P* = 0.335; *I*^2 ^= 82.5%) or laryngeal recurrent nerve injury rate (random effect: RR = 2.24 95% CI 0.93–5.39, *P* = 0.071; *I*^2 ^= 51.5%), chylothorax (fixed effect: RR = 0.34, 95% CI 0.11–1.02, *P* = 0.055; *I*^2 ^= 0%), arrhythmia(fixed effect: RR = 0.72, 95% CI 0.35–1.46, *P* = 0.360; *I*^2 ^= 0%) and mortality(fixed effect: RR = 0.75, 95% CI 0.16–3.58, *P* = 0.722; *I*^2 ^= 0%) in the VAME group compared with the VATE group (Figure 6, Table 3).

### Sensitivity analysis and publication bias

We did not perform sensitivity analysis and funnel plots because the number of included researches was less than 10.

## Discussion

As a newly developed surgical method, VAME has drawn a great body of attention since its first description in early 1990 (28). VAME overcomes the defects of visual field defects of blunt and blind operations in traditional transhiatal esophagectomy and enables surgeons to dissect the esophagus under direct vision though mediastinoscopy (29). Meanwhile, it adapts to patients in weak physical conditions, such as those combined with cardiopulmonary disease or aging patients to reduce postoperative complications (30). Previous reports have declared that this new approach has clinical advantages over the VATE approach. Considering that only a scarce number of studies with relatively limited sample sizes have been published, the evidence is patchy and the conclusion unclear. Therefore, we conducted this meta-analysis to comprehensively determine the strengths and weaknesses of VAME compared to VATE in esophageal cancer and try to provide solid evidence. To our knowledge, this is the first comprehensive meta-analysis on this topic.

In our meta-analysis, we included a total of eight studies, of which 350 esophageal cancer patients underwent VAME and 383 patients underwent VATE. This meta-analysis revealed that the VAME approach significantly shortened the operation time. Since surgery could be conducted more smoothly without having to change patients' positions in the VAME approach and could also be conducted by two teams simultaneously, while the thoracic segment of esophagus has to be loosened in a lateral position and the neck and abdominal approach could only be conducted in a supine position in the VATE approach. In order to reduce the heterogeneity, we did not included studies which compared robot-assisted transmediastinal esophagectomy with VATE for the much difference between robot-assisted and video-assisted surgery.

However, the VAME group retrieved less total lymph nodes than the VATE group. Lower thoracic mediastinal and abdominal lymph node dissection during the VAME approach were possible and not different compared to the VATE approach. Owing to the limited space and vision in the mediastinum, lymph node dissection in the middle mediastinum especially around the tracheal bifurcation was much more difficult. This revealed the defect of a less radical option for thoracic esophageal cancer due to view limitations and insufficient mediastinal lymphadenectomy compared with VATE (30). Lymph node metastasis along the recurrent laryngeal nerve is common in esophageal cancer and its dissection is of significance to improve long-term outcomes (31). Hence, some surgeons have suggested that VAME is suitable for patients without obvious enlargement of mediastinal lymph nodes. For patients with early-stage esophageal cancer, VAME can achieve parallel therapeutic effects.

Regarding postoperative complications, no difference was observed in common complications after esophagectomy. The VAME approach may resulted in relatively high recurrent laryngeal nerve injury rate or hoarse in the surgeons' early learning period. As summarized by Jin YX and colleagues (22), manipulation close to the esophagus and compression or stretching of adjacent tissues by instruments lead to lesion and edema of nerve tissues in the VAME approach. Furthermore, the overexposing laryngeal nerve affects the local blood supply to nerves and resulted in a high incidence of hoarseness. But after pooling the results, no significant difference was observed in the rate of recurrent laryngeal nerve injury rate. This may be owing to the proficiency of the surgeons after the initial learning period.

Pleural integration was usually retained in VAME surgery, which improved lung function compared to the VATE approach and reduced the influence on the lung and heart. On the other hand, patients underwent VAME may experience less chest pain after surgery, which makes it possible for patients to expectorate and exercise effectively in the early postoperative period. However, the pooled results revealed no difference of postoperative pulmonary complications and pneumonia between the two groups. As Feng MX et al. has noted (21), pulmonary complications were a kind of major problem after esophagectomy, and preserving the function of respiratory muscles and less pain resulting from a smaller incision could be beneficial in preventing pulmonary complications but the high rate of recurrent laryngeal nerves injury would exert adverse effects on patients receiving VAME. The two opposite effects could partly explain why no difference was observed in postoperative pulmonary complications between groups.

VAME alters the traditional surgical approach, and transthoracic operation is avoided, which is believed to play a significant role in reducing chest injury and maintaining the integrity of the thoracic cavity. Patients with poor cardiopulmonary functions unsuitable for thoracic surgery could now stand for trans-mediastinal surgery, because one-lung ventilation is omitted (30). From the traditional impression, patients who undergo VAME may have poor pulmonary function and be older. From our meta-analysis, no significant difference in age was observed in the VAME group compared with the VATE group (*P* = 0.955), the results of which were consistent with each single study. Two studies reported preoperative pulmonary function, and forced expiratory volume in one second (FEV1) and FEV1/forced vital capacity were not different between groups partly because of the difference in the study disign (19, 21).

Several limitations existed in our meta-analysis. First of all, as a complex operation, outcomes of which were significant association with the surgeon's techniques, clinical heterogeneities among the studies could also affect the validity of our result, and the operation type in the VATE group was also different which inevitably increased the clinical heterogeneity. Moreover, as a new technology, this approach has not been widely applied, and only a limited number of studies with small sample sizes could be obtained for analysis, which reduced the statistical power. Furthermore, there were much difference with regard to study design and outcomes definitions populations, so the internal heterogeneity was a big obstacle to interpret the results. Last but not least, esophageal cancer treatment has changed dramatically over the time period, particularly with respect to the standardized use of induction therapy for locally advance disease, therefore confounding factors are almost certainly present.

## Conclusion

In this meta-analysis, we compared the short-term outcomes and clinical pathological features in esophageal cancer patients receiving VAME to those receiving VATE. The results revealed that the VAME approach could significantly shorten operation time, but retrieved less lymph nodes. Intro- and postoperative complications were not different between the two groups. Further prospective studies with larger sample sizes are needed to confirm and update our results.

## Data Availability

The original contributions presented in the study are included in the article/Supplementary Material, further inquiries can be directed to the corresponding author/s.

## References

[1] Torre LA Bray F Siegel RL Ferlay J Lortet-Tieulent J Jemal A. Global cancer statistics, 2012. CA Cancer J Clin. (2015) 65:87–108. 10.3322/caac.21262 25651787

[2] Tran GD Sun XD Abnet CC Fan JH Dawsey SM Dong ZW Prospective study of risk factors for esophageal and gastric cancers in the linxian general population trial cohort in China. Int J Cancer. (2005) 113:456–63. 10.1002/ijc.20616 15455378

[3] Chan KKW Saluja R Delos Santos K Lien K Shah K Cramarossa G Neoadjuvant treatments for locally advanced, resectable esophageal cancer: a network meta-analysis. Int J Cancer. (2018) 143:430–7. 10.1002/ijc.31312 29441562

[4] Mariette C Piessen G Triboulet JP. Therapeutic strategies in oesophageal carcinoma: role of surgery and other modalities. Lancet Oncol. (2007) 8:545–53. 10.1016/S1470-2045(07)70172-9 17540306

[5] Luketich JD Pennathur A Awais O Levy RM Keeley S Shende M Outcomes after minimally invasive esophagectomy: review of over 1000 patients. Ann Surg. (2012) 256:95–103. 10.1097/SLA.0b013e3182590603 22668811 PMC4103614

[6] Tonutti M Elson DS Yang GZ Darzi AW Sodergren MH. The role of technology in minimally invasive surgery: state of the art, recent developments and future directions. Postgrad Med J. (2017) 93:159–67. 10.1136/postgradmedj-2016-134311 27879411

[7] Li B Xiang J Zhang Y Li H Zhang J Sun Y Comparison of Ivor-Lewis vs Sweet esophagectomy for esophageal squamous cell carcinoma: a randomized clinical trial. JAMA Surg. (2015) 150:292–8. 10.1001/jamasurg.2014.2877 25650816

[8] Wu B Xue L Qiu M Zheng X Zhong L Qin X Video-assisted mediastinoscopic transhiatal esophagectomy combined with laparoscopy for esophageal cancer. J Cardiothorac Surg. (2010) 5:132. 10.1186/1749-8090-5-132 21194430 PMC3022595

[9] Navarro-Ripoll R Cordova H Rodriguez-D'Jesus A Boada M Rodriguez de Miguel C Beltran M Cardiorespiratory impact of transesophageal endoscopic mediastinoscopy compared with cervical mediastinoscopy: a randomized experimental study. Surg Innov. (2014) 21:487–95. 10.1177/1553350613517943 24435021

[10] Yin Q Liu H Song Y Zhou S Yang G Wang W Clinical application and observation of single-port inflatable mediastinoscopy combined with laparoscopy for radical esophagectomy in esophageal squamous cell carcinoma. J Cardiothorac Surg. (2020) 15:125. 10.1186/s13019-020-01168-1 32503651 PMC7275589

[11] Okumura H Uchikado Y Matsumoto M Omoto I Sasaki K Kita Y Clinical significance of mediastinoscope-assisted transhiatal esophagectomy in patients with esophageal cancer. Langenbeck's Arch. Surg. (2015) 400:699–706. 10.1007/s00423-015-1330-y 26252999

[12] Yamagata Y Saito K Hirano K Oya M. Long-term outcomes and safety of radical transmediastinal esophagectomy with preoperative docetaxel, cisplatin, and 5-fluorouracil combination chemotherapy for locally advanced squamous cell carcinoma of the thoracic esophagus. World J Surg Oncol. (2020) 18:252. 10.1186/s12957-020-02023-2 32962718 PMC7510302

[13] Wang QY Li JP Zhang L Jiang NQ Wang ZL Zhang XY. Mediastinoscopic esophagectomy for patients with early esophageal cancer. J Thorac Dis. (2015) 7:1235–40. 10.3978/j.issn.2072-1439.2015.07.20 26380740 PMC4522484

[14] Wang J Jiang NQ Jiang B Wang ZL Zhang XY. Mediastinoscopy-assisted oesophagectomy in T1 oesophageal cancer patients with serious comorbidities: a 5-year long-term follow-up. Interact Cardiovasc Thorac Surg. (2015) 20:477–81. 10.1093/icvts/ivu433 25535180

[15] Yoshimura S Mori K Ri M Aikou S Yagi K Yamagata Y Comparison of short-term outcomes between transthoracic and robot-assisted transmediastinal radical surgery for esophageal cancer: a prospective study. BMC Cancer. (2021) 21:338. 10.1186/s12885-021-08075-1 33789620 PMC8010980

[16] Sugawara K Yoshimura S Yagi K Nishida M Aikou S Yamagata Y Long-term health-related quality of life following robot-assisted radical transmediastinal esophagectomy. Surg Endosc Other Interv Tech. (2020) 34:1602–11. 10.1007/s00464-019-06923-7 31286253

[17] Yoshimura S Mori K Yamagata Y Aikou S Yagi K Nishida M Quality of life after robot-assisted transmediastinal radical surgery for esophageal cancer. Surg Endosc Other Interv Tech. (2018) 32:2249–54. 10.1007/s00464-017-5918-x PMC5897479 29497828

[18] Guo L Zhao Q Wang K Zhao D Ye X Li T. A case-control study on the therapeutic effect of mediastinoscope-assisted and thoracoscope-assisted esophagectomy. Surg Innov. (2021) 28:316–22. 10.1177/1553350620958265 32909910

[19] Koide N Takeuchi D Suzuki A Miyagawa S. Mediastinoscopy-assisted esophagectomy for esophageal cancer in patients with serious comorbidities. Surg Today. (2012) 42:127–34. 10.1007/s00595-011-0042-3 22068678

[20] Nomura T Matsutani T Hagiwara N Fujita I Nakamura Y Makino H Mediastinoscopy-assisted transhiatal esophagectomy for esophageal cancer: a single-institutional cohort study. Surg Laparosc Endosc Percutan Tech. (2016) 26:E153–6. 10.1097/SLE.0000000000000348 27846176

[21] Feng MX Wang H Zhang Y Tan LJ Xu ZL Qun W. Minimally invasive esophagectomy for esophageal squamous cell carcinoma: a case-control study of thoracoscope versus mediastinoscope assistance. Surg Endosc Other Interv Tech (2012) 26:1573–8. 10.1007/s00464-011-2073-7 22179461

[22] Jin Y Lu X Xue L Zhao X. Retrospective comparison of two minimally invasive esophagectomy in the treatment of esophageal cancer: pneumatic mediastinoscopy versus thoracoscopy. J Laparoendosc Adv Surg Tech (2019) 29:638–42. 10.1089/lap.2018.0512 30562122

[23] Shi K Qian R Zhang X Jin Z Lin T Lang B Video-assisted mediastinoscopic and laparoscopic transhiatal esophagectomy for esophageal cancer. Surg Endosc Other Interv Tech. (2021) 36:7–4214. 10.1007/s00464-021-08754-x 34642798

[24] Wang QY Tan LJ Feng MX Zhang XY Zhang L Jiang NQ Video-assisted mediastinoscopic resection compared with video-assisted thoracoscopic surgery in patients with esophageal cancer. J Thorac Dis. (2014) 6:663–7. 10.3978/j.issn.2072-1439.2014.06.29 24976988 PMC4073377

[25] Liu W Guo X Zhao H Yu X Wang C Du L Mediastinoscopy-assisted transhiatal esophagectomy versus thoraco-laparoscopic esophagectomy for esophageal cancer: a single-center initial experience. J Thorac Dis. (2020) 12:4908–14. 10.21037/jtd-20-1328 33145064 PMC7578494

[26] Stang A. Critical evaluation of the Newcastle-Ottawa scale for the assessment of the quality of nonrandomized studies in meta-analyses. Eur J Epidemiol. (2010) 25:603–5. 10.1007/s10654-010-9491-z 20652370

[27] Egger M Davey Smith G Schneider M Minder C. Bias in meta-analysis detected by a simple, graphical test. BMJ. (1997) 315:629–34. 10.1136/bmj.315.7109.629 9310563 PMC2127453

[28] Buess G Becker HD. Minimally invasive surgery in tumors of the esophagus. Langenbecks Arch Chir Suppl II Verh Dtsch Ges Chir. (1990):1355–60. 1983542

[29] Bumm R Hölscher AH Feussner H Tachibana M Bartels H Siewert JR. Endodissection of the thoracic esophagus. Technique and clinical results in transhiatal esophagectomy. Ann Surg. (1993) 218:97–104. 10.1097/00000658-199307000-00015 8328835 PMC1242906

[30] Mimatsu K Oida T Kawasaki A Aramaki O Kuboi Y Kanou H Mediastinoscopy-assisted esophagectomy is useful technique for poor surgical-risk patients with thoracic esophageal cancer. Surg Laparosc Endosc Percutan Tech. (2009) 19:e17–20. 10.1097/SLE.0b013e31818aa5cc 19238050

[31] Tsurumaru M Kajiyama Y Udagawa H Akiyama H. Outcomes of extended lymph node dissection for squamous cell carcinoma of the thoracic esophagus. Ann. Cardiothorac. Surg. (2001) 7:325–9. 11888470

